# Editorial: OMICS-Based Approaches in Sports Research

**DOI:** 10.3389/fmolb.2022.870728

**Published:** 2022-05-20

**Authors:** Mohamed A. Elrayess, Francesco Botrè, Amelia Palermo

**Affiliations:** ^1^ Biomedical Research Center, QU Health, Qatar University, Doha, Qatar; ^2^ REDDs—Research and Expertise in Antidoping Sciences, ISSUL—Institute of Sport Science, University of Lausanne, Lausanne, Switzerland; ^3^ Department of Molecular and Medical Pharmacology, David Geffen School of Medicine, UCLA, Los Angeles, CA, United States

**Keywords:** omics, sports research, predictive biomarkers, anti doping, excercise

Genomics, transcriptomics and metabolomics are increasingly generating key insights for the development of precision approaches in heath and disease. Omics technologies have unlocked the high-throughput discovery of diagnostic biomarker and the systems-level evaluation of the efficacy and toxicity of novel therapies.

In the subfield of sport medicine and sport science, the application of -omics methods is relatively recent; yet, the number of peer-reviewed original research articles has seen a steady rise over the last years.

Athletic performance can be affected by a combination of endogenous and exogenous factors, including genomic and metabolomic profiles, as well as diet, medicament/drug intake, training regimen, and exercise. In this respect, the application of omics approaches encompassed, for instance, the multi-parametric assessment of training efficacy, injury predisposition, and the identification of robust biomarkers for the indirect detection of potentially “invisible” forms of doping. In addition, multi-omics integration approaches in sport medicine and doping sciences hold a clear promise for better understanding of the molecular/system levels of athlete’s pathophysiology.

What we believe should be indispensable to maximize the potential benefit that such approaches promise, is the promotion of cross-disciplinary and collaborative science, beyond the individual laboratories and research institutions.

If we wish to ensure that evidence-based, multi-omics studies, will be complementing efficiently, omics data and the associated meta-data should be available without delay in curated repositories. The availability of omics data generated in independent studies, will enable results validation in the context of distinct populations of subjects/athletes (e.g., biomarker validation), and allow to gauge unprecedented insight from genome-wide association studies (GWAS) to identify associations between genotypes and phenotypes and how this reflects on performance and drug testing. Meta analyses of literature data would indeed allow to assess and possibly validate any cause-effect relationships emerged from new studies, also, but not only, allow replication and confirmation of the results.

In this first volume of the special research topic “OMICS-Based Approaches in Sports Research”, we welcomed submissions tackling 1) integrated omics in sport physiology and pathophysiology, 2) predictive biomarkers associated with improved athletic performance and on sport related risks on athlete’ heath and injuries, 3) investigation of hormones and growth factors for improved anti-doping tests, 4) identification of novel biomarkers for the detection of sport doping, 5) novel statistical/modelling approaches for omics data integration, interpretation and doping detection. The feedback we received was beyond our most optimistic expectations: 57 scientists responded to our call with eight full length, high quality original articles. All published contributions are now available “open access.”

These studies covered different subfields of sport medicine and physiology of exercise, ranging from the interaction between mitochondrial function and the microbiome, to novel, indirect methods for the detection of doping.

Khoramipour et al. applied metabolomics to compare metabolic profiles and movement patterns between different male basketball player positions, with focus on relationships between indicators of internal and external loads during elite basketball games. The integration of video-based time motion analysis (VBTMA) with metabolomics of saliva samples and multivariate data analysis demonstrated distinct metabolic profiles of backcourt and frontcourt players, with significant changes involving aerobic/anaerobic pathways.

Mach et al. addressed the impact of endurance exercise on the functionality of mitochondria and on the composition of the gut microbiome in elite horses. The authors used transcriptomics, metabolomics and fecal microbiome analysis before and after hose endurance race. Their results show the association between energy metabolism, oxidative stress and inflammation with butyrate-producing bacteria, suggesting the possibility of enhancing/improving athletic performance by targeting the gut-mitochondria axis.

Narduzzi et al. used steroidomics and complete blood count analysis to tackle one among the most debated topics in doping testing, that is the detection of the intake of low doses of human growth hormone (GH) in association with recombinant erythropoietin. GH intake affects human hematopoiesis and steroidogenesis. Thus, the authors analyzed the complete blood count and the steroidomics profile in healthy, physically active young males and demonstrated that leukopoyetic and steroidal biomarkers in conjunction with classical endocrine biomarkers allow the accurate detection of the intake of micro-doses of recombinant GH.

Al-Muraikhy et al. dissected the influence of age and high intensity endurance on the effect of aerobic exercise on the complement system. Using peripheral blood, they show that high endurance elite athletes exhibit lower levels of complement components C2, C3b/iC3b and adipsin, and this observation is age-independent.

Al-Menhali et al. studied the proteomic responses of the heart to exercise training in mice by high resolution mass spectrometry. Proteomics analysis revealed specific changes in proteins involved in the respiratory electron transport chain and implicated in glutathione conjugation. These results represent a further step towards the full understanding of the molecular mechanisms underlying the beneficial effects of exercise on the heart.

Al-Muraikhy et al. addressed the issue of biological aging and its correlation to alterations in the metabolic pathways, specifically characterizing the metabolic activity in relation to telomere length in elite soccer players.

Lima et al. investigated whether whole blood in long-term storage and whole blood left over from standard hematological testing in short-term storage could be used for transcriptomic analysis, despite lacking RNA preservation; their result show that quantity, purity, and integrity were not significantly compromised from short- or long-term storage in blood storage tubes lacking RNA stabilization, indicating that transcriptomic analysis could be conducted using anti-doping samples collected or biobanked in absence of RNA preservation.

Finally, the first volume of this special research topic was closed by a perspective article by Sellami et al. outlining the state of the art and the expected forthcoming developments of molecular big data and omics-disciplines in sports sciences.

We thank all the Authors for the time and energy dedicated to the preparation and submission of the articles published in “OMICS-based Approaches in Sport Research”, and we look forward to welcome submissions of new articles for the second volume.

